# Supplementary material for: A rapid theta network mechanism for flexible information encoding
Source: Nat Commun. 2023 May 19;14:2872. doi: 10.1038/s41467-023-38574-7 (PMC10198978; doi:10.1038/s41467-023-38574-7)
Supplement: Supplementary file 1 — Supplementary Information [file 41467_2023_38574_MOESM1_ESM.pdf]

Supplementary information

Table S1. Individual patient information

	Age	Sex	Type	Correct CF trials	Correct CL trials	Re-referenced electrodes	Responsive electrodes
1	25	F	ECoG	30	26	49	10
2	30	M	ECoG	37	33	49	12
3	31	M	ECoG	39	40	57	21
4	25	M	ECoG	19	20	42	16
5	32	M	ECoG	30	22	43	20
6	41	M	sEEG	27	26	29	7
7	21	F	sEEG	26	25	33	10
8	27	M	sEEG	25	19	20	10
9	23	M	sEEG	36	30	80	27
10	27	M	sEEG	35	36	42	9
11	51	F	sEEG	25	22	5	3

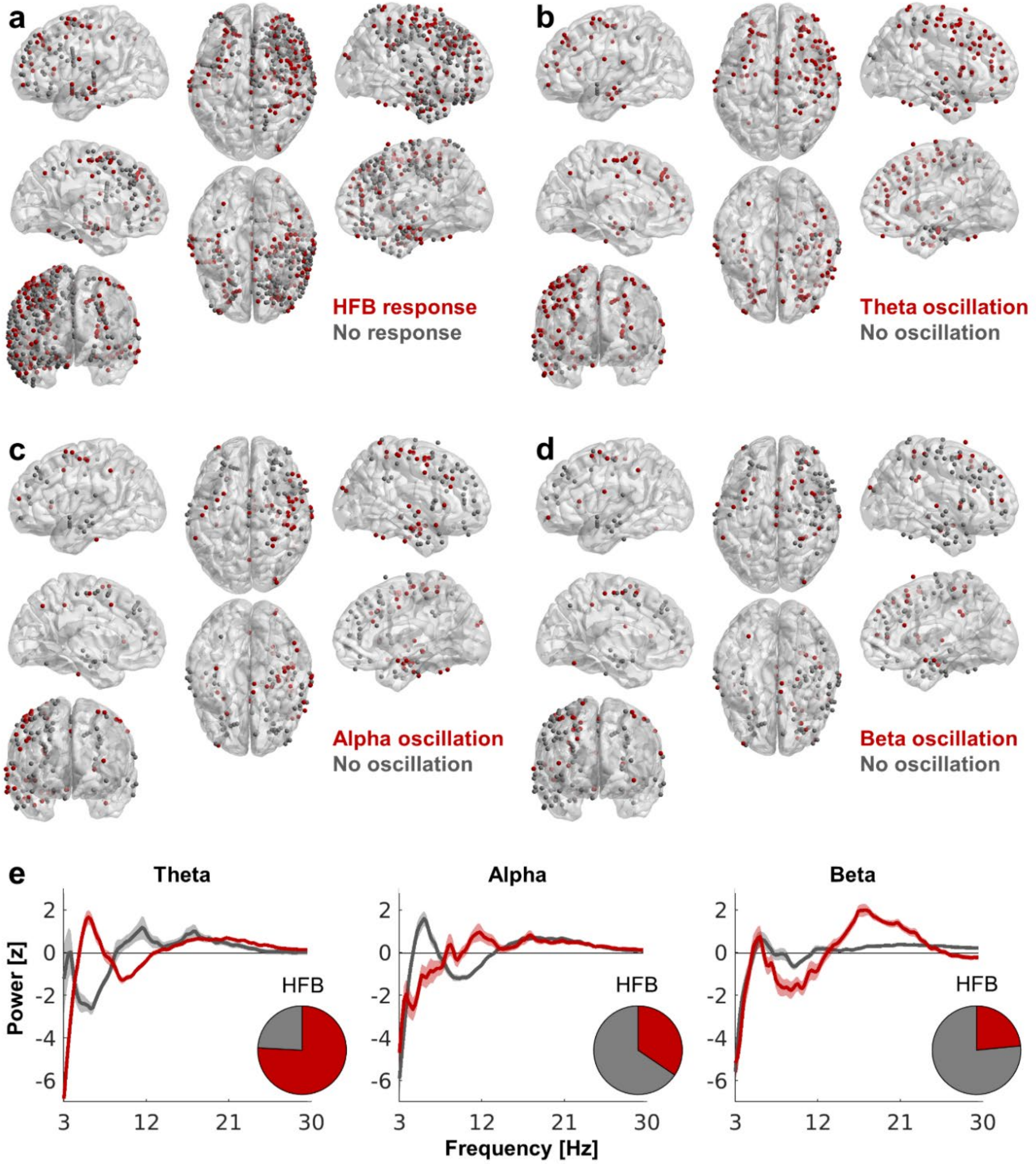

Figure S1. HFB and oscillatory characteristics of information encoding

a) Non-pathologic, artifact-free electrodes across all subjects overlaid on the MNI-152 template brain. Task-responsive electrodes included in subsequent analyses are shown in red. Non-responsive electrodes excluded from analyses are shown in gray. This figure was created using BrainNet Viewer[72].

b) Task-responsive electrodes across all subjects overlaid on the MNI-152 template brain. Oscillatory theta electrodes included in theta synchrony analyses are shown in red. Non-oscillatory electrodes excluded from theta synchrony analyses are shown in gray. This figure was created using BrainNet Viewer[72].

c) Same as (**b**) for oscillatory alpha electrodes included in alpha synchrony analyses.

d) Same as (**b**) for oscillatory beta electrodes included in beta synchrony analyses.

e) Power of task-responsive electrodes oscillating in the theta (**b**), alpha (**c**), and beta bands (**d**) after removing the aperiodic 1/*f* component. Oscillatory electrodes are shown in red. Non-oscillatory electrodes are shown in gray. Data are represented as M ± SEM, calculated across electrodes. Insets indicate the percentage of electrodes oscillating at each frequency out of all task-responsive electrodes.

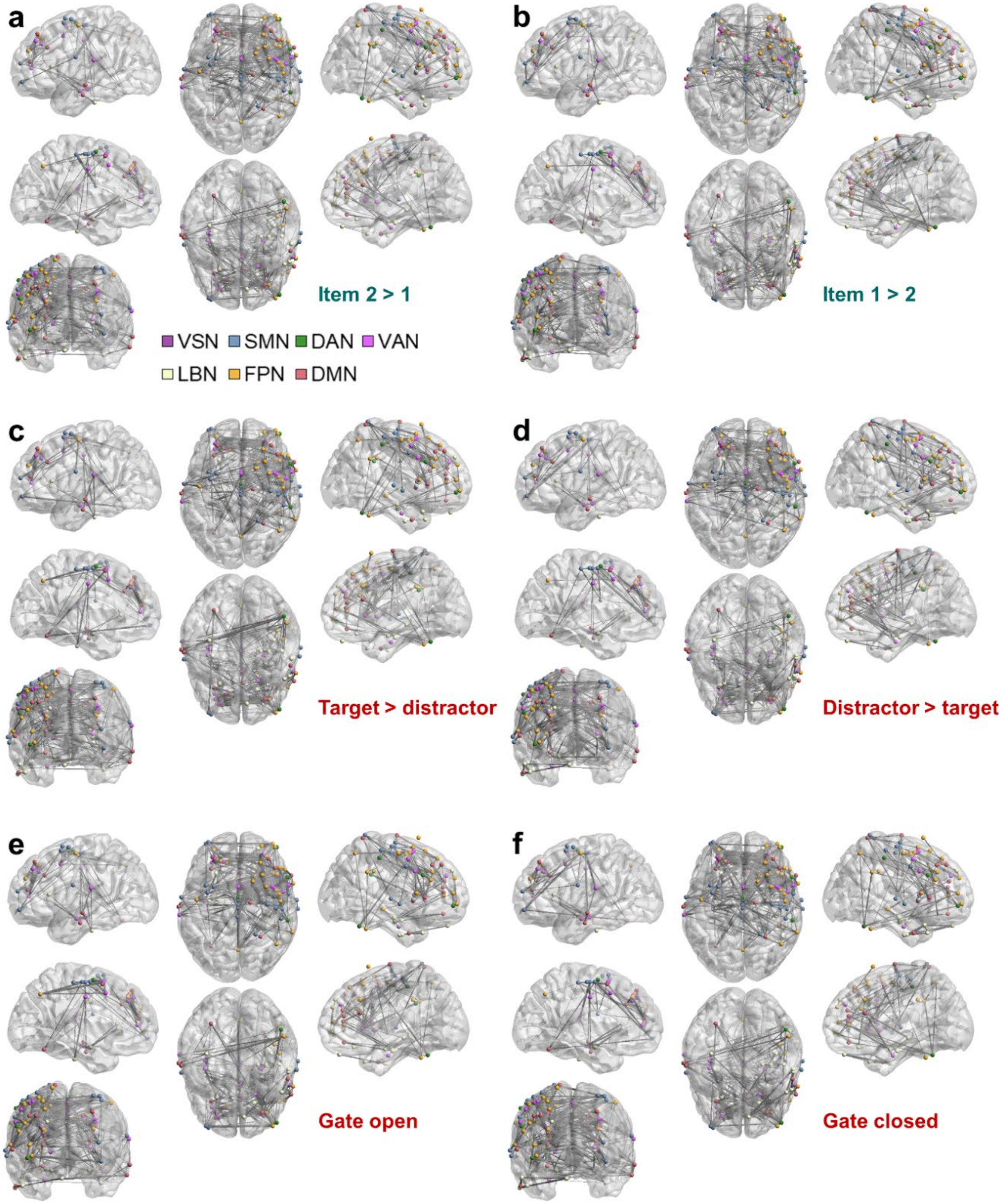

Figure S2. Theta synchrony effects by network

a) Theta synchrony difference scores on correct CL trials with item 2 > 1 synchrony, across all subjects overlaid on the MNI-152 template brain. The colors of electrodes indicate the network. Inter-electrode lines are shown in gray. Line thickness indicates the relative size of the item 2 > 1 difference ($0 < z < 0.45$). This figure was created using BrainNet Viewer[72].

b) Same as (**a**) on correct CL trials with item 1 > 2 synchrony. Line thickness indicates the relative size of the item 1 > 2 difference ($-0.28 < z < 0$).

c) Same as (**a**) on correct CF trials with target > distractor synchrony. Line thickness indicates the relative size of the target > distractor difference ($0 < z < 0.32$).

d) Same as (**a**) on correct CF trials with distractor > target synchrony. Line thickness indicates the relative strength of the distractor > target difference ($-0.27 < z < 0$).

e) Same as (**a**) on correct CF trials where target > distractor synchrony preceded faster RT. Line thickness indicates the relative size of the RT × (target > distractor) correlation ($-3.04 < z < 0$).

f) Same as (**a**) on correct CF trials where distractor > target synchrony preceded faster RT. Line thickness indicates the relative size of the RT × (distractor > target) correlation ($0 < z < 3.15$).

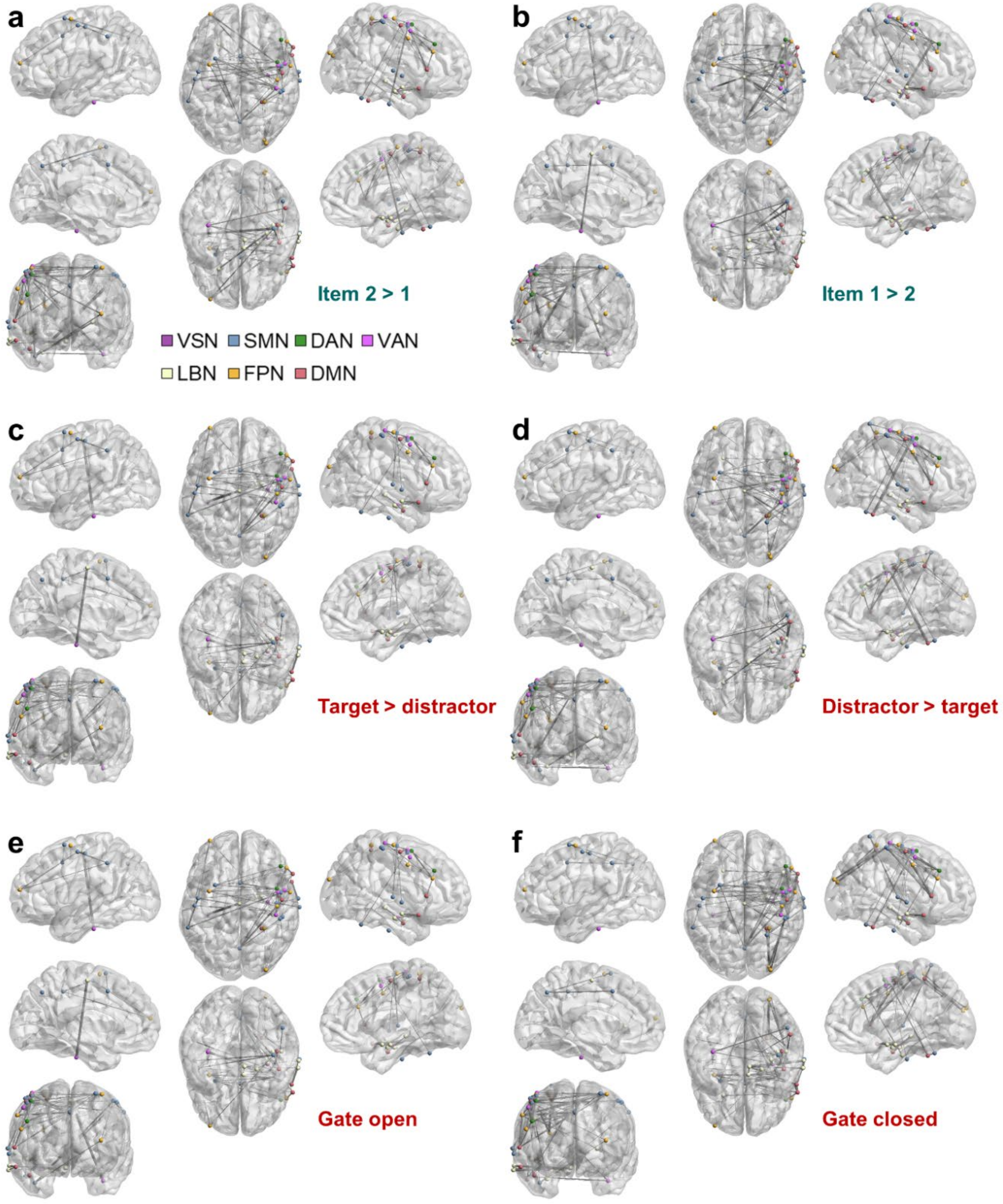

Figure S3. Alpha synchrony effects by network

a) Alpha synchrony difference scores on correct CL trials with item 2 > 1 synchrony, across all subjects overlaid on the MNI-152 template brain. The colors of electrodes indicate the network. Inter-electrode lines are shown in gray. Line thickness indicates the relative size of the item 2 > 1 difference ($0 < z < 0.21$). This figure was created using BrainNet Viewer[72].

b) Same as (**a**) on correct CL trials with item 1 > 2 synchrony. Line thickness indicates the relative size of the item 1 > 2 difference ($-0.13 < z < 0$).

c) Same as (**a**) on correct CF trials with target > distractor synchrony. Line thickness indicates the relative size of the target > distractor difference ($0 < z < 0.16$).

d) Same as (**a**) on correct CF trials with distractor > target synchrony. Line thickness indicates the relative strength of the distractor > target difference ($-0.17 < z < 0$).

e) Same as (**a**) on correct CF trials where target > distractor synchrony preceded faster RT. Line thickness indicates the relative size of the RT × (target > distractor) correlation ($-2.06 < z < 0$).

f) Same as (**a**) on correct CF trials where distractor > target synchrony preceded faster RT. Line thickness indicates the relative size of the RT × (distractor > target) correlation ($0 < z < 2.72$).

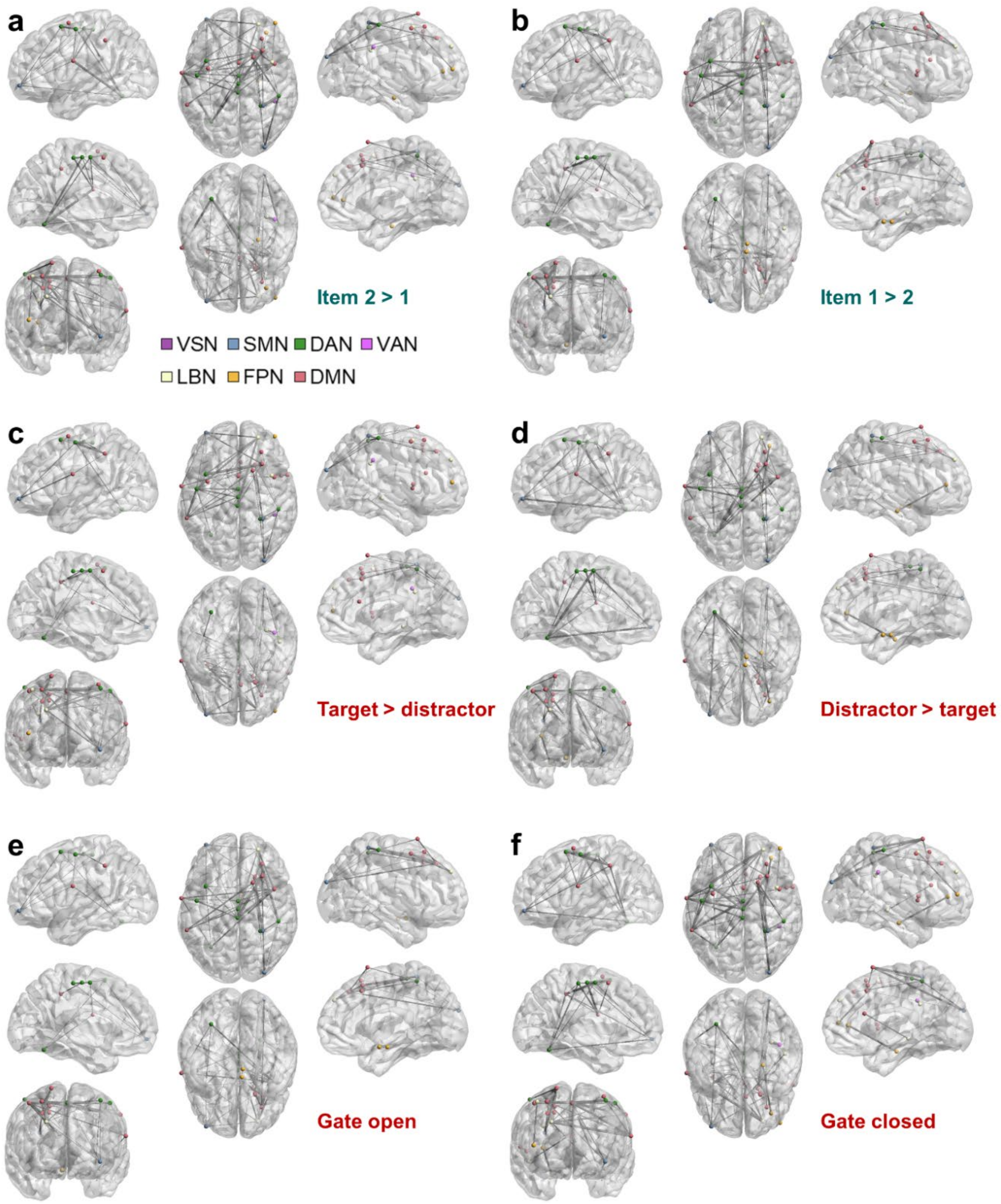

Figure S4. Beta synchrony effects by network

a) Beta synchrony difference scores on correct CL trials with item 2 > 1 synchrony, across all subjects overlaid on the MNI-152 template brain. The colors of electrodes indicate the network. Inter-electrode lines are shown in gray. Line thickness indicates the relative size of the item 2 > 1 difference ($0 < z < 0.10$). This figure was created using BrainNet Viewer[72].

b) Same as (**a**) on correct CL trials with item 1 > 2 synchrony. Line thickness indicates the relative size of the item 1 > 2 difference ($-0.11 < z < 0$).

c) Same as (**a**) on correct CF trials with target > distractor synchrony. Line thickness indicates the relative size of the target > distractor difference ($0 < z < 0.13$).

d) Same as (**a**) on correct CF trials with distractor > target synchrony. Line thickness indicates the relative strength of the distractor > target difference ($-0.13 < z < 0$).

e) Same as (**a**) on correct CF trials where target > distractor synchrony preceded faster RT. Line thickness indicates the relative size of the RT × (target > distractor) correlation ($-2.34 < z < 0$).

f) Same as (**a**) on correct CF trials where distractor > target synchrony preceded faster RT. Line thickness indicates the relative size of the RT × (distractor > target) correlation ($0 < z < 1.89$).
