# Supplementary material for: A rapid theta network mechanism for flexible information encoding
Source: Nat Commun. 2023 May 19;14:2872. doi: 10.1038/s41467-023-38574-7 (PMC10198978; doi:10.1038/s41467-023-38574-7)
Supplement: Supplementary file 3 — Reporting Summary [file 41467_2023_38574_MOESM3_ESM.pdf]

nature portfolio

Corresponding author(s): Elizabeth Johnson, David Badre

Last updated by author(s): Apr 5, 2023

Reporting Summary

Nature Portfolio wishes to improve the reproducibility of the work that we publish. This form provides structure for consistency and transparency in reporting. For further information on Nature Portfolio policies, see our Editorial Policies and the Editorial Policy Checklist.

Statistics

For all statistical analyses, confirm that the following items are present in the figure legend, table legend, main text, or Methods section.

n/a	Confirmed	
☐	☒	The exact sample size (*n*) for each experimental group/condition, given as a discrete number and unit of measurement
☐	☒	A statement on whether measurements were taken from distinct samples or whether the same sample was measured repeatedly
☐	☒	The statistical test(s) used AND whether they are one- or two-sided<br>*Only common tests should be described solely by name; describe more complex techniques in the Methods section.*
☐	☒	A description of all covariates tested
☐	☒	A description of any assumptions or corrections, such as tests of normality and adjustment for multiple comparisons
☐	☒	A full description of the statistical parameters including central tendency (e.g. means) or other basic estimates (e.g. regression coefficient) AND variation (e.g. standard deviation) or associated estimates of uncertainty (e.g. confidence intervals)
☐	☒	For null hypothesis testing, the test statistic (e.g. *F*, *t*, *r*) with confidence intervals, effect sizes, degrees of freedom and *P* value noted<br>*Give P values as exact values whenever suitable.*
☐	☒	For Bayesian analysis, information on the choice of priors and Markov chain Monte Carlo settings
☐	☒	For hierarchical and complex designs, identification of the appropriate level for tests and full reporting of outcomes
☐	☒	Estimates of effect sizes (e.g. Cohen's *d*, Pearson's *r*), indicating how they were calculated

Our web collection on statistics for biologists contains articles on many of the points above.

Software and code

Policy information about availability of computer code

Data collection	Data were collected using custom-built MATLAB 8.6 (R2015b) scripts with the open-source Psychtoolbox 3.0.14 software extension.
Data analysis	Data were analyzed using custom-built MATLAB 9.7 (R2019b) scripts with the open-source FieldTrip (R20210301) software extension. Bayesian correlation was performed using the open-source JASP software (2020). BrainNet Viewer 1.7 was used to visualize electrodes and inter-electrode functional connections on the MNI-152 template brain.

For manuscripts utilizing custom algorithms or software that are central to the research but not yet described in published literature, software must be made available to editors and reviewers. We strongly encourage code deposition in a community repository (e.g. GitHub). See the Nature Portfolio guidelines for submitting code & software for further information.

Data

Policy information about availability of data

All manuscripts must include a data availability statement. This statement should provide the following information, where applicable:

- Accession codes, unique identifiers, or web links for publicly available datasets
- A description of any restrictions on data availability
- For clinical datasets or third party data, please ensure that the statement adheres to our policy

The data and codes used in this study are available in the OSF database under accession code https://osf.io/rx2zd.

Human research participants

Policy information about studies involving human research participants and Sex and Gender in Research.

Reporting on sex and gender	All subjects were adult neurosurgical patients undergoing intracranial EEG (iEEG) monitoring for seizure management, and sex was determined retroactively based on hospital records. Individual demographic data including sex and age are provided in Table S1. Data from 3 female and 8 male patients were included in this study. No sex-based analyses were performed due to low sample size.
Population characteristics	See above. Subjects were aged 21-51 years.
Recruitment	Subjects were neurosurgical patients at the University of California, Irvine, University of California, Davis, and California Pacific Medical Center hospitals, and not recruited specifically for this study.
Ethics oversight	All subjects provided written informed consent in accordance with the Declaration of Helsinki as part of the research protocol approved by the Institutional Review Board of the University of California, Irvine, University of California, Davis, or California Pacific Medical Center.

Note that full information on the approval of the study protocol must also be provided in the manuscript.

Field-specific reporting

Please select the one below that is the best fit for your research. If you are not sure, read the appropriate sections before making your selection.

☒ Life sciences ☐ Behavioural & social sciences ☐ Ecological, evolutionary & environmental sciences

For a reference copy of the document with all sections, see nature.com/documents/nr-reporting-summary-flat.pdf

Life sciences study design

All studies must disclose on these points even when the disclosure is negative.

Sample size	Eleven human neurosurgical patients participated. This sample size was driven solely by the availability of patients, a rare sample. This sample matches the sizes seen in many iEEG studies.
Data exclusions	Subjects were included in the study based on pre-established criteria: above-chance behavioral performance (< 0.35 errors, chance 0.5), iEEG sampling of frontal cortex, no major lesions. Artifactual trials and channels were removed from the iEEG data to ensure that effects would not be driven by noise or pathology.
Replication	Behavioral results were compared to a published study using the same experimental task in non-clinical samples (Chatham et al., 2014), and found to be consistent. iEEG analyses were performed per subject and identified predictors of behavioral outputs in all 11 subjects.
Randomization	N/A - the within-subjects design meant that all subjects took part in all conditions.
Blinding	All data preprocessing routines and analyses of task-responsive electrodes and oscillatory electrodes were performed blinded to experimental condition and iEEG electrode placements.

Reporting for specific materials, systems and methods

We require information from authors about some types of materials, experimental systems and methods used in many studies. Here, indicate whether each material, system or method listed is relevant to your study. If you are not sure if a list item applies to your research, read the appropriate section before selecting a response.

Materials & experimental systems

n/a	Involved in the study
☒	☐ Antibodies
☒	☐ Eukaryotic cell lines
☒	☐ Palaeontology and archaeology
☒	☐ Animals and other organisms
☒	☐ Clinical data
☒	☐ Dual use research of concern

Methods

n/a	Involved in the study
☒	☐ ChIP-seq
☒	☐ Flow cytometry
☒	☐ MRI-based neuroimaging
